# Characterization of Bacterial Communities in Volcanic Soil from Northern Patagonian Area of Chile

**DOI:** 10.3390/microorganisms13112519

**Published:** 2025-11-01

**Authors:** Patricia Aguila-Torres, Mauricio González, Marcela Hernández, Constanza Aguado-Norese, Jonathan E. Maldonado, Richard M. Miranda, Roxana González-Stegmaier, Daniel E. Palma, Luis A. Rojas, Macarena Mellado

**Affiliations:** 1 Laboratorio de Microbiología Molecular, Escuela de Tecnología Médica, Universidad Austral de Chile, Sede Puerto Montt, Av. Los Pinos s/n Balneario Pelluco, Puerto Montt P.O. Box 1327, Chile; 2 Laboratorio de Bioinformática y Expresión Génica, Instituto de Nutrición y Tecnología de los Alimentos, Universidad de Chile, Santiago 7830490, Chile; mgonzale@inta.uchile.cl (M.G.); mconstanza.aguado@gmail.com (C.A.-N.); daniel.palma.i@ug.uchile.cl (D.E.P.); 3 Millennium Institute Center for Genome Regulation (MI-CGR), Santiago 8320165, Chile; 4 School of Biological Sciences, University of East Anglia, Norwich NR4 7TJ, UK; marcela.hernandez@uea.ac.uk; 5 Laboratorio de Multiómica Vegetal y Bioinformática, Departamento de Biología, Facultad de Química y Biología, Universidad de Santiago de Chile, Santiago 9170022, Chile; jonathan.maldonado@usach.cl; 6 Millenium Institute of Integrative Biology (iBio), Santiago 7500565, Chile; 7 Laboratorio de Biología Molecular, Instituto de Gestión e Industria, Universidad Austral de Chile, Puerto Montt P.O. Box 1327, Chile; richard.miranda@uach.cl; 8 Translational Medicine Laboratory, Centro de Investigación e Innovación en Cáncer. Fundación Arturo López Pérez OECI Cancer Center, Santiago 7500921, Chile; roxana.gonzalez@falp.org; 9 Departamento de Química, Facultad de Ciencias, Universidad Católica del Norte, Antofagasta 1200000, Chile; l.rojas@ucn.cl; 10 Centro Integrativo de Biología y Química Aplicada (CIBQA), Facultad de Ciencias de la Salud, Universidad Bernardo O’Higgins, Santiago 8370854, Chile; macarena.melladom@gmail.com

**Keywords:** microbial communities, volcanic soils, Northern Patagonia, Osorno volcano

## Abstract

Osorno volcano (41.1° S, 72° W) is located in the Andean Southern Volcanic Zone. The volcano lies within a national park as part of the protected areas system. This setting provides an opportunity to compare soil microbial communities between sectors with (H) and without (NI) anthropogenic activities within a volcanic territory. To do so, we selected one of the most visited volcanoes in Chilean Patagonia to examine composition, diversity (taxonomic and phylogenetic), and co-presence and mutual exclusion interaction networks between members of volcanic soil bacterial communities. Soil DNA was extracted, and the 16S rRNA gene was analyzed by high-throughput DNA sequencing, followed by taxonomic identification. The most prevalent phylum across all sites (H and NI) was *Pseudomonadota*, followed by *Acidobacteriota*, *Actinobacteriota*, and *Chloroflexota*. Based on taxonomic and phylogenetic indices, we found that the diversity of bacteria was significantly less in the humanized area than in the non-intervened areas. Beta diversity analysis also revealed a clear separation between humanized and non-intervened soils. Additionally, a decrease in network connectivity was observed at NI sites. Our results provide clear evidence that anthropogenic factors, such as tourism, vehicle parking, and combustion processes, are key drivers shaping bacterial community structure in volcanic soils, with potential consequences for ecosystem health and the capacity to provide ecosystem services.

## 1. Introduction

Ecological integrity and resilience of ecosystems to future environmental changes are essential elements to ensure human well-being [1] yet they are vulnerable to increasing anthropogenic pressures such as land use change, livestock, deforestation, recreation, sports, and tourism [2]. It has been proposed that soil communities differ depending on the state of soil degradation, responding to both the physicochemical properties of the soil and the degree of anthropogenic intervention [3,4,5].

Microorganisms are among the first colonizers of volcanic deposits [6] and play an essential role in volcanic soil ecosystems [7]. Volcanic habitats are colonized by microorganisms capable of fixing nitrogen and carbon from the atmosphere [8]. These extreme environments are generally complex habitats due to their high exposure to UV radiation, temperature fluctuations, and pH [8]. Furthermore, microbial communities are highly adapted to these environments, with carbon monoxide serving as an alternative carbon and energy source for the colonizing microorganism of volcanic substrates [6,9,10].

Studies on the microbial community structure of young volcanic deposits have shown that these were influenced by colonizing plants [11]. Some factors that contribute to volcanic microbial diversity are pH, elemental composition, minerals, temperature, water content, and pressure [8]. Inside the available metals content from soil, such as zinc [11] and iron [12], at lower concentrations, are essential micronutrients for microorganisms. In young, unvegetated sites, early colonizers of volcanic soils face high ultraviolet radiation (UV), oxidative stress, and highly variable temperatures [8]. Microbial succession on newly exposed, unvegetated substrates is a crucial component in the initial stages of biogeochemical cycling and ecosystem development. The introduction of plants, however, significantly modifies the composition of the pioneer microbial community [13]. This modification occurs through the input of carbon in the form of root exudates and litter deposits. These carbon inputs from plants facilitate further development of the ecosystem. Consequently, plant colonization plays a pivotal role in shaping early microbial communities and advancing ecosystem maturation [14].

Regarding taxonomic composition, previous studies of bacterial communities of volcanoes located at the volcanic zone of southern Chile have revealed the predominance of members belonging to *Ktedonobacteria* (the *Chloroflexi* phylum), which are able to oxidize hydrogen and carbon monoxide, and are found mainly in non-vegetated soils [6,15]. In addition, early bacterial communities have been reported to grow on volcanic ash deposits derived from the 2000 eruption of Mount Oyama on Miyake Island in Japan [16] and the 2015 eruption at Calbuco Volcano in Chile [17]. Anderson et al., 2022 [7] reported that the most prevalent phyla across all sites at Tatun Volcano Group, Northern Taiwan, were *Pseudomonadota*, *Actinobacteriota*, *Acidobacteriota*, and *Chloroflexota*. In the Krafla area in Iceland, according to 16S rRNA gene amplicon sequencing, the most abundant phyla were *Proteobacteria*, *Acidobacteria*, *Actinobacteria*, and *Firmicutes* [18]. Young and older volcanic soils differ in several ways, including microbial communities, soil composition, and physical properties [19]. For example, older volcanic soils have greater microbial richness, diversity, and evenness than younger soils, but younger soils have a more complex microbial network [19].

The Southern volcanic zone of the Andes, that extends from 33° to 46° S, is shaped with at least 60 historically and potentially active volcanoes in Chile and Argentina, including the Osorno volcano (2652 m. above sea level), a composite stratovolcano of Mid-Pleistocene age [20]. mainly composed by basaltic to basaltic-andesite products [21,22]. This historically active volcano is located within the Vicente Pérez Rosales National Park, near populated areas of Chile, in the Los Lagos region, where areas near tourist facilities, parking lots, and other more protected areas coexist.

This comparative approach between humanized and non-intervened sites highlights the ecological significance of understanding how tourism and other human activities may affect microbial diversity and, consequently, the resilience and functioning of volcanic ecosystems. In addition, other comparative studies on soil bacterial communities in a site with and without human intervention, such as agricultural practices, have shown significant and complex changes in soil bacterial communities [23,24]. A common effect is the reduction in bacterial diversity (alpha index), often linked to factors like pesticide application or soil management. However, frequent anthropogenic disturbance can, paradoxically, increase total bacterial abundance. Crucially, this intervention alters the community composition, changing the relative abundance of dominant phyla and potentially enriching pathogens, reflecting how land use directly modifies nutrient levels and soil structure. Therefore, there are no studies of microbial diversity in the Osorno volcano, so its basic knowledge is still limited and represents an opportunity to assess soil diversity, taxonomic composition, and assembly of bacterial communities in volcanic areas with and without anthropogenic activities in Northern Patagonia, Chile.

## 2. Materials and Methods

### 2.1. Site Description and Collection of Volcanic Soil Samples

Vicente Pérez Rosales National Park (41°06′ S 72°30′ O) covers an area of 253,568 hectares and is located between Llanquihue and “Todos los Santos” lakes of Chile [20,25]. In terms of flora, highlighted species include tepa (*Laureliopsis philippiana*), mañío (*Podocarpus nubigenus*), coigüe (*Nothofagus dombeyi*), ulmo (*Eucryphia cordifolia*), tiaca (*Caldcluvia paniculata*), and luma (*Amomyrtus luma*). A total of 18 soil samples were collected in January 2022 from six distinct sites on Osorno Volcano, categorized into two main groups based on presumed human impact: three Humanized (H) sites near tourist facilities and three Non-Intervention (NI) sites with restricted access. While the study was designed to assess the effects of human activity, the H and NI areas exhibited significant inherent natural differences, particularly in vegetation cover (largely absent in H) and underlying soil composition (e.g., organic matter, N, C, and trace elements) (Figure S1, Table S1). These sites correspond to areas near tourist facilities and parking zones located at the base of the Osorno Volcano, which experience frequent human activity, vehicle movement, and soil compaction, but lack vegetation cover. In contrast, the non-intervened (NI) sites were selected from restricted-access areas within the national park, covered by native vegetation and showing no visible signs of anthropogenic disturbance. Briefly, 5 cm of topsoil was removed, and then ~1 kg of the underlying soil (15 cm of depth) was placed in a sterile glass jar and stored at 4 °C until processing [26]. Samples were taken using a square pattern (1 m^2^) and triplicate samples were taken at each point, with a total of nine replicates per site (humanized and non-intervened).

### 2.2. Physico-Chemical Soil Analysis

The collected samples were analyzed as composite samples for physical properties and chemical soil quality parameters. Soil carbon (C), nitrogen (N), organic matter, electrical conductivity (EC), and pH were measured following methods established by the Normalization and Accreditation Commission (CNA) of the Chilean Society of Soil Science [27]. Additionally, heavy metals in volcanic soil were measured, including arsenite (As), cadmium (Cd), chromium (Cr), copper (Cu), iron (Fe), lead (Pb), manganese (Mn), nickel (Ni), mercury (Hg), molybdenum (Mo), and zinc (Zn). These were quantified using atomic absorption spectrophotometry (AAS), following the procedures outlined by [28]. Selenium (Se) was also measured following methods established by the Normalization and Accreditation Commission (CNA) of the Chilean Society of Soil Science [27] (Tables S2 and S3).

### 2.3. DNA Extraction from Volcanic Soil Samples

Volcanic soil DNA was extracted as described previously [28]. Briefly, the DNA was extracted from soils starting from 3 g of volcanic samples. These samples were resuspended in 3 mL extraction buffer (1% (*w*/*v*) CTAB, 1.5 M NaCl, 100 mM Tris–HCl; pH 8, 100 mM Na_2_HPO_4_, 100 mM Na EDTA; pH 8). After that, 10 mg/mL of lysozyme was added to the sample, mixed briefly, and then incubated at 37 °C for 1 h. Then, samples were centrifuged at room temperature using 6000× *g* for 10 min. The obtained supernatant was then applied as sample input for the DNeasy PowerSoil Pro Kit (QIAGEN) following the manufacturer’s instructions. A Qubit fluorimeter (ThermoFisher Scientific, Vacaville, CA, USA) was used to quantify the final genomic DNA concentration. The integrity and purity of the DNA were checked by agarose gel electrophoresis.

### 2.4. 16S-rRNA Amplicon Sequencing

PCR amplicon libraries for bacterial 16S rRNA gene were prepared using primers flanking the V3–V4 region of the 16S rRNA (primers 341F/806R). 16S rRNA gene amplification and sequencing were carried out. To amplify DNA, the primers 341F (5′-CCTAVGGGRBCCASCAG-3′) and 806R (5′-GGACTACNNGGGTATCTAAT-3′) were used, and 16S rRNA gene sequencing was done using the Illumina PE250 platform at Novogene, UK [17].

### 2.5. Sequencing Data Analysis

16S rRNA gene sequence analysis was carried out for samples from two areas (H and NI) in triplicate (post sequencing, the NI1.3 sample was deleted because its number of reads was below the rarefaction threshold). Raw 16S ribosomal amplicon sequences were processed and filtered with protocols described by [29,30] using the software Mothur v.1.22.2 [31]. The filtered sequences were analyzed using Quantitative Insights into Microbial Ecology software (QIIME v2-2023.2) [32] with default parameters. Representative sequences were taxonomically classified against the SILVA r16S database (v138) [33] using the sklearn method of QIIME [34]. To avoid the high number of uninformative low-expression/low-representation Amplicon Sequence Variants (ASVs) we selected reads with at least a 0.01% abundance for analyses. The ASV enrichment probability was calculated using a linear statistical model on relative abundance as reported by [35], and the ASVs’ differential abundance was calculated using a Bayes moderated *t*-test [36]. Venn diagrams of ASVs across samples were diagrammed using the online tools Venny [37] and JVenn [38].

### 2.6. Microbial Diversity Analysis

Microbial diversity based on ASVs sequences were analyzed using previously described methods [39] using the QIIME software suite (QIIME v2-2023.2) [32]. From the final data matrix, the observed number of species (Richness), Shannon index (H’), Faith phylogenetic diversity index (PD), and Good’s Coverage index were calculated. There were calculated rarefaction curves for each metric, serial subsampling each column to a standardized 33,000 sequences per sample. To evaluate beta diversity, the software MEGAN Community Edition v6.21.1 [40] was used, generating Principal Coordinates Analysis (PCoA) and Unweighted Pair Group Method with Arithmetic Average (UPGMA) tree with Bray-Curtis distance at phylum and genus taxonomic levels. Microbial functions profiles were predicted based on the 16S rRNA gene using PICRUSt [41].

### 2.7. Statistical Analysis

The similarity and distance of the samples were evaluated using classical clustering with multivariate analysis, as described previously [42], and implemented in PAST software (v4.06).

To investigate the association between environmental variables and microbial community composition at the family level, Canonical Correspondence Analysis (CCA) was performed using the software package PAST version 3.22. The ASV abundance matrix was log-transformed to stabilize variances. To ensure comparability, environmental variables were standardized to a zero mean and unit variance [43,44].

Three statistical analyses were used to identify families with significant differences in abundance between sample groups: RNAseq y, metagenomeSeq, and LEFse. ASVs were considered significant if they had significant differences in at least two tests. These analyses were performed using MicrobiomeAnalyst software v2.0 [45].

To evaluate the differentially abundant predicted functions between H and NI samples, a Welch’s test was conducted on the abundance of KO genes using the software package PAST version 3.22.

### 2.8. Network of the Microbial Communities

Regarding the pipeline for each network: ASVs that were present in at least five samples were selected. In order to address the compositional characteristics of the data [46], a centered log-ratio transformation was applied, adding a pseudocount of 1 to address zero values. NetCoMi [47] was employed to construct co-occurrence networks. The Pearson correlation matrix was computed, and a minimum correlation threshold was determined using the R package RMThreshold v1.1 [48]. NetCoMi was then used to calculate the network layouts with the signed transformation (assigning an edge weight of 1 for a correlation of 1 and a weight of 0 for a correlation of −1) and to identify clusters using the fast greedy algorithm. Finally, the co-occurrence network was protted in Cytoscape v3.8.2 [49], which also provided network statistics.

## 3. Results

### 3.1. Soil Characteristics

A general characterization of the volcanic soils was performed by analyzing their physicochemical properties and composition. The pH values of the volcano soil samples were close to or equal to 7.0 at both locations (Table S2). Regarding the humanized soils, they presented low organic matter (OM) (0.1–0.89%) and carbon content (0.06–0.52%) compared with non-intervened soils (Table S2). Similarly, regarding the heavy metals present in the volcanic soils, our results showed an average concentration for the H-NI soil samples of 0.1–0.208 mg kg^−1^ for As, 0.557–0.683 mg kg^−1^ for Cd, 6.62–45.4 mg kg^−1^ for Cu, 1.101–1.73 mg kg^−1^ for Cr, 2154.6–3038 mg kg^−1^ for Fe, 32.3–52 mg kg^−1^ for Mn, 0.06–0.102 mg kg^−1^ for Hg, 4.49–4.40 mg kg^−1^ for Mo, 4.82–5.93 mg kg^−1^ for Ni, 0.49–4.37 mg kg^−1^ for Pb, 0.015–0.016 mg kg^−1^ for Se, 5.12–4.79 mg kg^−1^ for Zn, respectively. The analysis of the soluble fraction of these elements revealed slightly higher values in H samples compared to NI samples (Table S3). Notably, zinc exhibited a higher concentration in NI samples compared with H samples (Table S3). However, despite the observed variations, the differences between H and NI areas were not statistically significant (Kruskal-Wallis test, *p* = 0.71).

### 3.2. Soil Bacterial Community Composition

**Figure 1:**
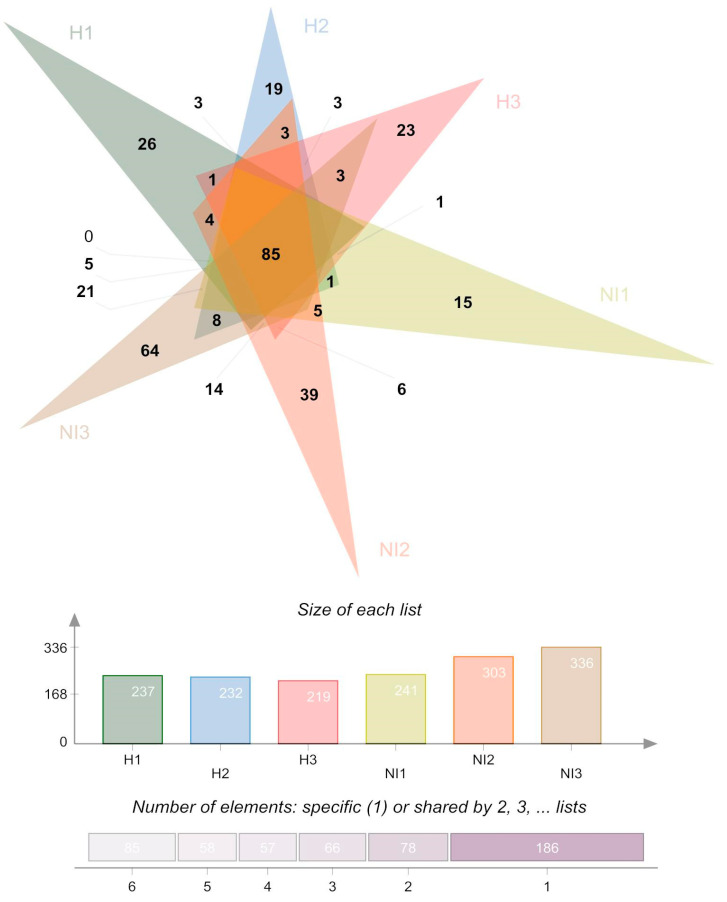
Venn diagram of the ASVs, at the genus level, present in each sample (all genera from humanized and non-intervened sites) by averaging the replicates. The numbers in the Venn diagram represents the number of ASVs that belongs to each intersection.

We obtained 953,041 reads through 16S rRNA gene amplicon sequencing analysis, which led to 28,718 ASVs detected from humanized and non-intervened samples (Table 1 and Table S4). When performing a preliminary cluster analysis of samples, sample NH1.3 from site NH1 clustered with samples from site NH3. To avoid further problems, we decided to discard this sample. Therefore, site NH1 was analyzed with only two sample replicates. The taxonomic composition of volcanic soil samples comprised eight phyla (Table S5). A Venn diagram was determined by all ASVs from volcanic soil samples, including an intersection at the genus level, which shows that there are 85 shared genera (Figure 1). These taxa correspond to the genera, *WPS-2*, *AD3*, *IMCC26256*, and *Candidatus Udaeobacter* with prevalence values > 70%. The ASV core (ASVs detected in all corresponding samples) for H was 26 genera that accumulated on average 13.80% of the abundance. On the other hand, the ASV core for NI was 37 ASVs that accumulated on average 8.81% of the abundance (Figure 1).

**Figure 2:**
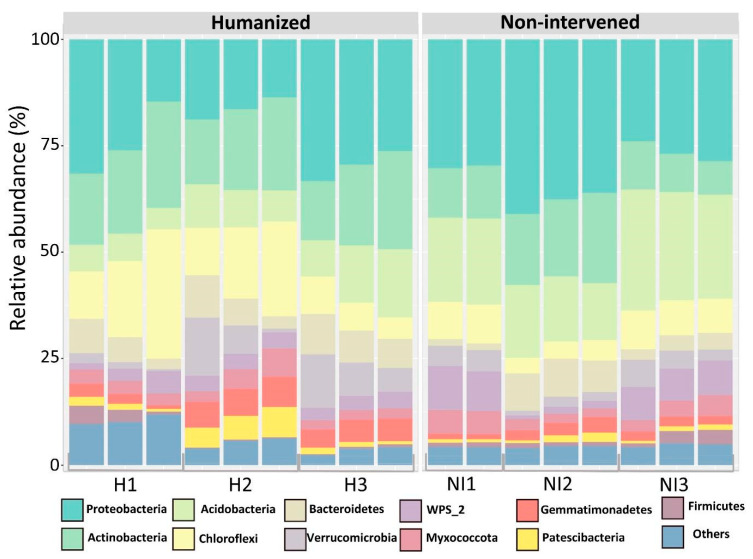
Relative abundance of main phyla in humanized (H) and non-intervened (NI) samples. The dominant phyla are those accounting for more than 90% of the total relative abundance across all samples. Phyla with a lower relative abundance are grouped under the category “Others”.

From the ASVs, 11 phyla were identified from the humanized sites and 12 from the non-intervened samples. From the humanized sites, five phyla, constituting 73% of the total relative abundance, were identified, whereas in non-intervened samples, 73.2% of the relative abundance belonged to four phyla. Unclassified reads and low relative abundance phyla (less than 1%) were classified as others and ranged between 2.7 and 6.1% in NI and H, respectively (Figure 2). The total relative abundance showed that the most abundant phyla in H samples were *Proteobacteria* (23.6%), *Actinobacteriota* (19.3%), *Chloroflexi* (14.4%), *Acidobacteriota* (9.2%), and *Bacteriodota* (6.6%). On the other hand, the most abundant phyla in NI samples were *Proteobacteria* (31.7%), *Acidobacteriota* (21%), *Actinobacteriota* (13.3%), and *Chloroflexi* (7.2%) (Figure 2).

The relative abundance of the phylum *Chloroflexi* doubled for H samples compared to NI samples, same for *Gemmatimonadota*, *Acidobacteriota*, *WPS-2*, and *Patescibacteria* (Figure 2).

To study the most representative microbial groups in volcanic soil, we decided to analyze a subset of the data with a relative abundance greater than 1% (Table S5). At the class level, the highest relative abundance was observed for Alphaproteobacteria and Betaproteobacteria in the H and NI samples (Table S5).

**Figure 3:**
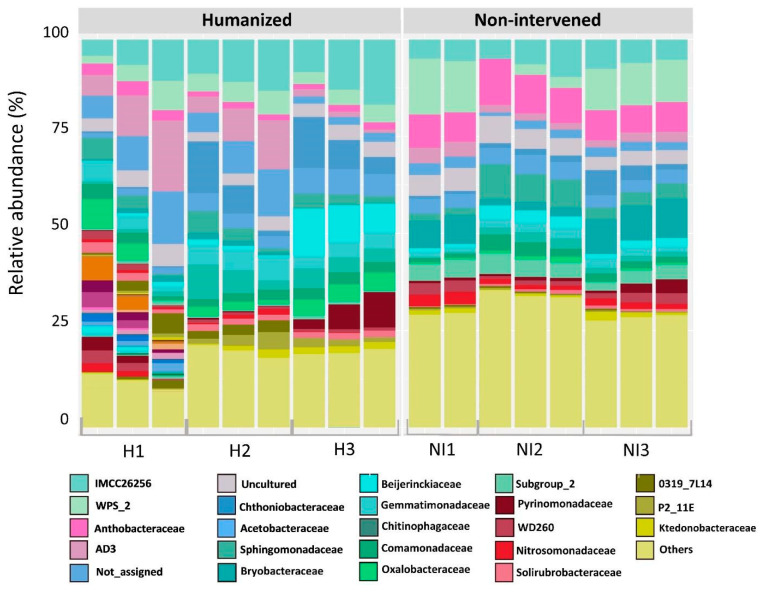
Relative abundance at the family level in humanized (H) and non-intervened (NI) samples. Low-abundance families (less than 1%) are shown as “others”.

At the family level, the bacterial community also varied between the two geographical areas (humanized and non-intervened). From all families within the H and NI samples, 212 and 230 were observed, respectively. Twenty-four families were obtained in the humanized site (representing 63.3% of the total community), and 22 families in the non-intervened samples (representing 58.9% of the total community). Unclassified ASVs and low relative abundance families (less than 1%) classified as others fluctuated between 41.1% and 36.7% in NI and H, respectively (Table S5). According to these analyses, total relative abundance showed that, within the H samples, the most abundant families were *IMCC26256* (7.4%), *AD3* (6%), *Chthoniobacteraceae* (5.3%), *Gemmatimonadaceae* (4.5%), *Oxalobacteraceae* (3.7%), *Chitinophagaceae* (3.6%), *Beijerinckiaceae* (3.6%), *WPS-2* (3.4%), *Acetobacteraceae* (3.3%), *Shingomonadaceae* (2.3%), *Comamonadaceae* (2.2%), *Pyrinomonadaceae* (2.1%), *Xanthobacteraceae* (2%), *Solirubrobacteraceae* (1.9%), P2-11E (1.6%), *Hymenobacteraceae* (1.3%), *Subgroup_7* (1.3%), *Blastocatellaceae* (1.3%), *Anaeromyxobacteraceae* (1.2%), 0319-7L14 (1.1%), *WD260* (1.1%), *Saccharimonadales* (1.0%), *Solibacteraceae* (1.0%), (Figure 3). On the other hand, families in the NI samples, belong mainly to the families *Xanthobacteraceae* (6.5%), *WPS-2* (6.2%), *Bryobacteraceae* (6.1%), *IMCC26256* (4.1%), *Subgroup_2* (3.4%), *Shingomonadaceae* (3.4%), *Acetobacteraceae* (3.1%), *Chitinophagaceae* (3%), *Chthoniobacteraceae* (2.4%), *Comamonadaceae* (2.4%), AD3 (2.3%), *Gemmatimonadaceae* (2.2%), *Pyrinomonadaceae* (1.7%), *Nitrosomonadaceae* (1.7%), *Thermoanaerobaculaceae* (1.6%), *Solibacteraceae* (1.5%), *Beijerinckiaceae* (1.4%), *Polyangiaceae* (1.3%), *WD260* (1.3%), *Pseudonocardiaceae* (1.1%), and *Ktedonobacteraceae* (1.1%) (Figure 3).

### 3.3. Alpha and Beta Diversity

**Figure 4:**
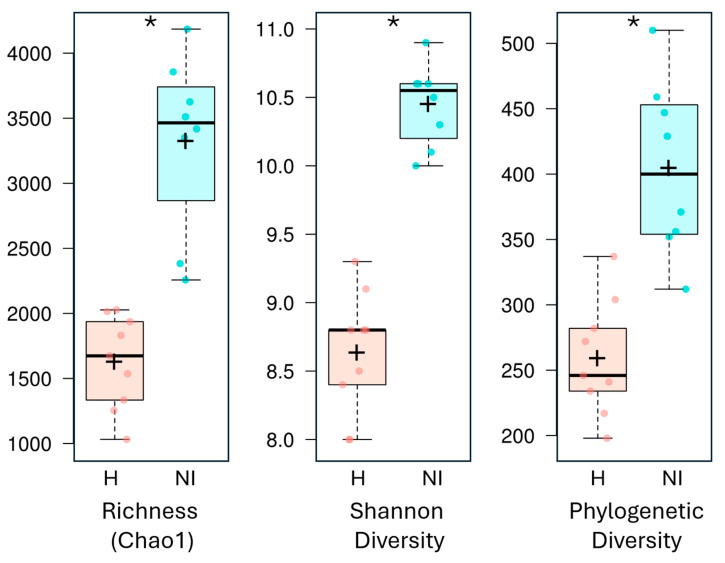
Alpha diversity composition between humanized and non-intervened samples. From left to right, it shows richness (Chao index), diversity (Shannon index) and phylogenetic diversity (PD). The lower and upper edges of the box represent the 25th and 75th percentiles, respectively, while the line inside the box indicates the median. The whiskers extend to the limits of the distribution determined by the upper and lower quartiles. Individual dots represent samples. An asterisk (*) indicates statistical significance based on the Kruskal-Wallis pairwise comparison (H-NI) (*p* > 0.05).

Next, we analyzed the microbial diversity and composition in seventeen samples from two areas: humanized and non-intervened. The comparison of bacterial diversity (Phylogenetic Diversity and Shannon index) and richness (Total ASVs and Chao index) allowed us to evaluate alpha diversity (Table 1). Statistical analysis identified significant differences between humanized and non-intervened sites regarding total ASVs, Chao1, Shannon index, and phylogenetic diversity. Specifically, the average of ASVs (richness) was 1521 and 3159 in H and NI sites, respectively. Similarly, the Chao1 index was 1627 and 3323 in humanized and non-intervened sites, respectively. The Shannon index and Phylogenetic Diversity were 8.6 and 259, respectively, in humanized areas, compared to 10.5 and 405 in non-intervened areas. In addition, within alpha diversity indices, those from non-intervened areas were significantly higher than those in humanized areas (Mann-Whitney test *p* < 0.05) (Figure 4). Our results indicate higher bacterial diversity in both soil types of the Osorno volcano compared to Llaima volcano [6]. In the Llaima study, Shannon index values ranged from 5.21 in younger soils to 6.99 in older soils. By contrast, the Osorno soils demonstrated higher values, ranging from 8.0 to 10.9 (Table 1).

**Figure 5:**
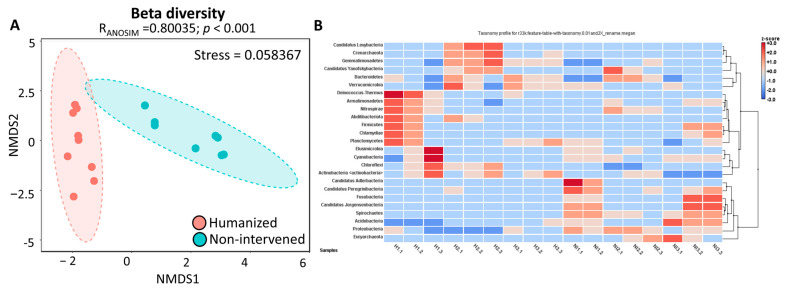
Beta diversity and composition of humanized and non-intervened samples. (**A**) Nonmetric multidimensional scaling (NMDS) plots using the Bray-Curtis dissimilarity index and ANOSIM test for humanized and non-intervened samples. The ANOSIM test shows the R-statistic (R) and the statistical significance (*p* < 0.001). (**B**) Heatmap of the relative abundance of bacterial phyla. Columns represent samples, and rows represent phyla with more than 1% relative abundance in at least one sample. The colors of the columns indicate the abundance. Column clustering was performed using the Bray–Curtis distance and the complete clustering method. H samples: humanized; NI samples: non-intervened.

For beta diversity, a nonmetric multidimensional scaling (NMDS) analysis and ANOSIM test were employed to assess differences in microbial community composition between humanized and non-intervened soils. The NMDS plot revealed a clear separation between the two areas, indicating distinct microbial assemblages (Figure 5A). The ANOSIM test further confirmed this distinction, with a global R-value of 0.85 (*p* < 0.001), signifying strong dissimilarity between humanized and non-intervened soils.

Additionally, an analysis comparing taxonomic composition between humanized and non-intervened areas was conducted, along with a hierarchical clustering based on Bray-Curtis distance to analyze the entire bacterial communities of both site types. The data revealed two differentiated clusters. One cluster contains all volcanic soil samples of the humanized site (H samples) and 3 out of 8 non-intervened samples (NI2.1, NI2.2, NI2.3; green color). The other cluster contains the remaining NI samples (NI1.1, NI1.2, NI3.1, NI3.2, NI3.3) (Figure S2 and Figure 5). These results suggest that common bacterial communities are lightly modulated by humanized samples, which tend to approximate bacterial communities in non-intervened sites.

### 3.4. Heatmap and Cluster Analysis

The relative abundances at the phylum level are shown as a heatmap in Figure 5B, where two main groups of subsampled rarefied libraries can be distinguished. Phyla that managed to be exclusive to the libraries in the first cluster are *Candidatus Levybacteria*, *Acrenarchaeota*, *Deinococcus thermus*, *Abditibacteriota*, and *Chloroflexi*.

### 3.5. Differential Abundance Analysis

Moreover, to determine which families are driving the differentiation between the H and NI samples, we performed a differential abundance analysis at the family level (Table S5). In this study, 105 families with different abundances between these two groups of samples were determined, of which 53 were more abundant in the NI samples and 52 in the H samples.

### 3.6. Potential Functional Diversity and Distribution Patterns of Functional Microbes

To explore the predicted functional capacities of the prokaryotic communities in the volcanic soils, PICRUSt2 was used. PICRUSt2 predicts the metabolic functions from the 16S rRNA gene sequences. The bacterial communities from the Osorno volcano were predominantly enriched in pathways associated with aminoacyl tRNA biosynthesis, carbon fixation pathway in prokaryotes, chaperones and folding catalyst, arginine and proline metabolism, pyruvate metabolism, transcription factors, ribosome biogenesis, DNA replication proteins, amino sugar and nucleotide sugar metabolism, glycine, serine and threonine metabolism, glycolysis/gluconeogenesis, lipid biosynthesis proteins, aminoacid related enzymes, Chromosome, bacterial motility proteins, peptidases, purine metabolism, ABC transporters, DNA repair and recombination proteins, Ribosome and transporters (Figure S3).

Beyond these universal housekeeping functions, we examined PICRUSt2 predictions for metabolic pathways with potential implications for volcanic soil ecosystems. Carbon metabolism included ribulose-bisphosphate carboxylase (rbcL, K01601) for carbon fixation and aerobic carbon monoxide dehydrogenases (coxL, coxM, coxS; K03518–K03520) for atmospheric CO oxidation. Sulfur oxidation was represented by the Sox system (soxA/X/B/Y/Z/C; K17222–K17227). Nitrogen cycling functions comprised periplasmic nitrate reductase (napA complex; K02567–K02572) and nitrite reductase (nirK, K00368). Phosphorus acquisition included the phosphonate utilization operon (phn genes; K05774–K06166) and acid phosphatases (appA, K01093). Additional functions included mycothiol biosynthesis (mshA, mshC, mshD; K15520–K15526), Type III (K03224, K03230) and Type VI secretion systems (K11891, K11893, K11900–K11907), stress response proteins such as uspB (K06144) and ahpC (K03386), and carotenoid enzymes (crtW, K09836; crtZ, K15746) (Table S6).

To identify differentially abundant KEGG orthologous genes (KOs) between H and NI samples, a differential abundance analysis was performed (Table S6). This analysis identified 1100 differentially abundant functions, of which 848 were enriched in NI samples and 252 in H samples. We then assessed whether any of these KOs had been previously reported as relevant to soil health. Among the 1100 differentially abundant KOs, 48 had been documented as important for key biogeochemical processes, specifically the nitrogen cycle, phosphate cycle, carbon cycle, and oxygen availability. Notably, all 43 of these soil-health-related KOs were enriched in NI samples and only five were enriched in H samples (Table S6).

NI samples showed enrichment in functions related to carbon fixation (rbcL), atmospheric CO oxidation (coxL/coxM/coxS), sulfur oxidation (soxA/X/B/Y/Z/C), periplasmic nitrate reduction (napA complex), phosphonate metabolism (phn operon), acid phosphatases (appA), and stress tolerance (uspB, ahpC). In contrast, H samples were enriched in carbohydrate metabolism (e.g., galK, K00849; endoglucanase, K01179; glycogen synthase, K00693), nitrite reduction (nirK, K00368), Type III/VI secretion systems (K03224, K03230; K11891, K11893, K11900–K11907), mycothiol biosynthesis (mshA/mshC/mshD; K15520/K15521/K15526), tryptophan biosynthesis (trpE/trpG; K01657/K01658), phosphoenolpyruvate carboxykinase (pckA, K01596), dihydrolipoamide dehydrogenase (DLD, K00382), metal homeostasis regulators (cueR, K19591; fur, K03711), carotenoid modification (crtW, K09836; crtZ, K15746), and metal/micronutrient ABC transporters (ABC.MN; K09819–K09820). Notably, H also showed enrichment for macrolide efflux (mef, K08217), mercury resistance (merC, K19058), and aromatic dioxygenases (hcaC, K05710), which are consistent with anthropogenic pressures (antibiotics, metals, and aromatic inputs).

### 3.7. Relative Contribution of Physicochemical Soil Characteristics to Bacterial Community Structure

**Figure 6:**
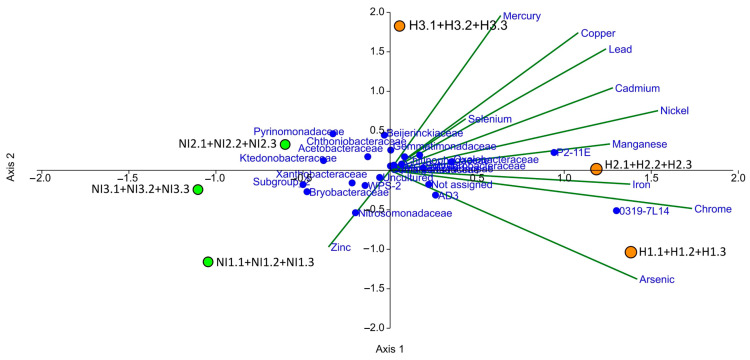
Ordination diagram of canonical correspondence analysis that shows relationship between the relative abundance of different bacterial communities whithin family taxa and metal variables.

Canonical correspondence analysis (CCA) was employed to examine how various physicochemical soil parameters contributed to the changes in bacterial community structure in NI and H samples of volcanic soils (Figure 6). Zinc is an essential micronutrient, it is absorbed by plants as a Zn^2+^ ion, acting as a metallic component of enzymes and participating in the metabolism of proteins and carbohydrates [50]. According to the CCA, NI samples were closely related to the vegetated volcanic soils. Concentration of Zn in these samples was relatively high (5.1 mg Kg^−1^) for this area (NI1.1, NI1.2, and NI1.3), unlike the Hg and As (0.06 and 0.1 mg Kg^−1^), which were more prevalent in H samples (0.102 and 0.1 mg Kg^−1^) (Figure 6). Among the major bacterial families in non-intervened areas, we found *Nitrosomonadaceae*, *Ktedonobacteraceae*, *Bryobacteraceae*, *Xanthobacteraceae*, Acetobacteraceae, *Chthoniobacteraceae*, and *Pyrinomonadaceae*. On the other hand, the family *Nitrosomonadaceae* was identified from the 16S rRNA gene, analyzing close to zinc (Figure 6), which are ammonia oxidizers that generally exert control over nitrification by oxidizing ammonia to nitrite, which is subsequently oxidized by bacterial nitrite oxidizers to nitrate [51].

### 3.8. Co-Occurrence Network Structure and Modularity in Volcanic Soils

#### 3.8.1. Overall Network Properties and Differential Connectivity of ASVs

**Figure 7:**
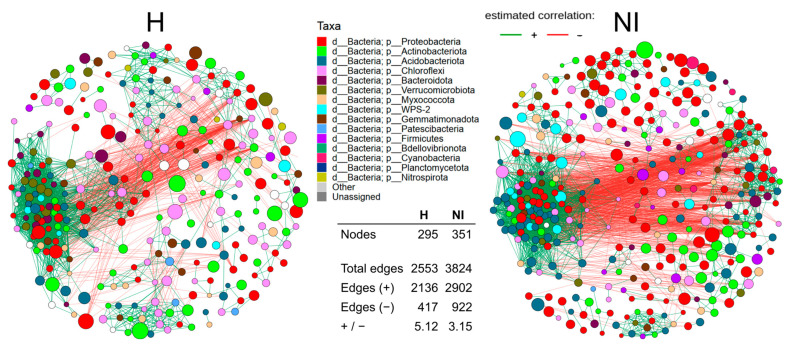
Co-occurrence networks of microbial communities in volcanic soils. Node size reflects the clr-transformed abundance of the ASV, and its color indicates the ASV’s phylum. Green lines show positive correlations, while red lines indicate negative correlations.

To explore potential interactions among microbial taxa in volcanic soils from humanized and non-intervened sites, we constructed co-occurrence networks for the two soil conditions (H and NI; Figure 7). In the networks, node colors represent the taxonomic affiliation at the phylum level, and node sizes represent their centered log-ratio (clr) transformed abundance.

The network corresponding to H soils consisted of 295 nodes and 2553 edges, whereas the NI network contained 351 nodes and 3824 edges. Combined, the two networks contained a total of 6377 edges, consisting of 5038 positive (co-presence) and 1339 negative (mutual exclusion) correlations. In both networks, positive associations dominated over negative ones, but the ratio differed markedly. In H soils, 2136 positive and 417 negative correlations were detected (+/− ratio = 5.12), while in NI soils, 2902 positive and 922 negative correlations were identified (+/− ratio = 3.15) (Table S7.1).

To further examine the higher positive-to-negative interaction ratio observed in the H network, we analyzed ASVs shared between both networks that exhibited the largest increases in this ratio. Five taxes accounted for a substantial portion of the change (Table S7.2 and Table S7.3). For example, *Sphingomonas* (*Proteobacteria*) shifted from a balanced profile in NI (19 positive and 19 negative interactions; ratio = 1.0) to exclusively positive associations in H (23/0; ratio = 23.0). Similarly, *JG30-KF-CM66* (*Chloroflexi*) and *WPS-2* showed more than fivefold increases in their positive-to-negative ratios, while *Blastocatella* (Acidobacteriota) and an additional *Sphingomonas* ASV also displayed pronounced increases. In most cases, these shifts were driven primarily by the loss or substantial reduction of negative associations in H rather than marked gains in positive links (Table S7.2 and Table S7.3).

#### 3.8.2. Modular Structure and Taxonomic Composition of Clusters

To assess the internal architecture of microbial co-occurrence networks, we applied modularity analysis to identify clusters, defined as subsets of ASVs more densely connected than to the rest of the network. In both networks, intra-cluster associations were predominantly positive (2556 positives and 36 negatives in NI; 1984 positives and 21 negatives in H), whereas inter-cluster associations were mainly negative (886 negatives and 346 positives in NI; 396 negatives and 152 positives in H) (Table S7.2 and Table S7.3).

The NI and H networks were partitioned into 29 and 31 clusters, respectively, based on topological community detection. These modules varied notably in size and taxonomic composition. In the NI network, the three largest clusters were cluster 7 with 70 ASVs, cluster 1 with 58, and cluster 3 with 37 (Table S7.2 and Table S7.3). Cluster 7 was dominated by *Proteobacteria*, *Actinobacteriota*, and *Acidobacteriota*, while cluster 1 was enriched in *Acidobacteriota* and *Proteobacteria*, and cluster 3 comprised mainly *Proteobacteria* and *Actinobacteriota*. In contrast, the H network was organized into slightly smaller clusters, with cluster 1 (56 ASVs), cluster 2 (53), and cluster 4 (43) as the largest. Cluster 1 contained *Proteobacteria*, *Acidobacteriota*, and *Bacteroidota*; cluster 2 included *Proteobacteria*, *Actinobacteriota*, and *Chloroflexi*; and cluster 4 was enriched in *Proteobacteria* and *Chloroflexi*. These compositional patterns indicate that while *Proteobacteria* remained dominant across conditions, *Acidobacteriota* were more abundant in NI, whereas *Chloroflexi* and *Bacteroidota* gained relative importance in H (Table S7.2 and Table S7.3).

#### 3.8.3. Potentially Keystone Taxa

To evaluate whether specific taxa might exert a central influence on the structural integrity of the networks, we identified potentially keystone taxa, defined here as taxa with high centrality relative to their abundance (“low-abundance, high-influence” nodes). Specifically, ASVs ranking within the top 10% of eigenvector centrality while occurring below the median relative abundance were considered. Eigenvector centrality was selected as it emphasizes nodes connected to other highly connected nodes, thereby highlighting ASVs central to overall network cohesion (Table S7.2 and Table S7.3).

In total, 17 potentially keystone taxa were identified in the NI network and 15 in the H network. The NI network harbored a greater phylogenetic breadth, including representatives of *WPS-2*, *Gemmatimonadota*, and several lineages of *Acidobacteriota*. Most of these ASVs were located in central modules, particularly cluster 1, which was highly connected. For example, members of Subgroup_12 and Subgroup_2 (*Acidobacteriota*) and uncultured *Gemmatimonadota* displayed high centrality values despite their low abundance. By contrast, the H network contained fewer and taxonomically less diverse potentially keystone taxa. These were primarily affiliated with *Actinobacteriota* and *Chloroflexi,* and they were positioned more peripherally in the network (Table S7.2 and Table S7.3).

## 4. Discussion

Soil properties and its microbiome are important to soil and plant health according to [3] within the topic of the “One Health” concept. Ecological integrity and the capacity of ecosystems to resist future environmental changes are essential elements to guarantee human well-being. However, they are vulnerable to increasing pressures of anthropogenic origin, such as land use change, livestock, deforestation, real estate development, recreational activities, and tourism (among others) [1]. In this scenario, it is essential to consider that ecosystem resilience can also be estimated by considering the evolutionary perspective of the communities hosted, which allows for showing complementary patterns to those provided by traditional biodiversity measurements [52]. In particular, it has been proposed that soil bacterial communities, plant roots, and their exudates differ depending on the state of soil degradation, responding both to the physical-chemical characteristics of the soil, as well as to the degree of anthropogenic intervention it presents [3]. In our results, the pH values of the volcano soil samples were close to or equal to 7.0 at both locations, which were within the previously described range for Chilean volcanic soils [53]. Notably, zinc exhibited a higher concentration in NI samples compared with H samples, which could indicate that Zn participates in plant metabolism [54]. With respect to taxonomy, the most prevalent phylum across all sites (H and NI) was *Pseudomonadota*, followed by *Acidobacteriota*, *Actinobacteriota*, and *Chloroflexota*. These results are in agreement with those reported by [55], in which 16S rRNA gene analysis revealed that the predominant microbial communities in volcanic soils of Chiloé (South of Chile) and in soils of Andean temperate forests in Chile (with varying levels of anthropogenic degradation) were *Proteobacteria*, *Acidobacteria*, and *Actinobacteria*. Moreover, previous analyses performed in Volcanic soils from Chile and specifically from the LLaima volcano in Southern Chile [6,55], showed that the analysis of bacterial 16S rRNA genes displayed high abundances of *Proteobacteria*, *Actinobacteria*, *Acidobacteria*, and *Chloroflexi* in the eruption of 1640 and 1751, which coincides with the main phyla found in our study.

In contrast, our results identified low abundances of the phyla *Planctomycetes* (0.46%) and *Verrucomicrobia* (4.68%) that were found in the 1957 eruption [6]. Although we are not identifying bacterial communities after an eruption, the results of these investigations show us a baseline of the communities that have been identified in volcanic soils in southern Chile. The differences in our results with respect to those found by them could be because the LLaima volcano has a high abundance of *Planctomycetes*, and these have been associated with basaltic communities in soils formed by lava [6], and it is an active volcano that has been rebuilding its microbiome. In addition, the young soil communities of Hawaiian volcanic deposits (1959-KVD) were dominated by *Acidobacteria*, *Alpha*- and *Gammaproteobacteria* (*Proteobacteria*), *Actinobacteria*, *Cyanobacteria*, and many unclassified phylotypes [56], The first three phyla agree with our results (more abundant), as did the NI sites with more vegetation compared to the H sites, which presented higher diversity indices.

The *Chloroflexi* phylum was a common factor in volcanic soils from Chile, and these results agree with the analysis of the microbial communities in the soils of the Llaima volcano in Chile [6]. Hernández et al., (2020b) [10] suggested that volcanic soil from Chile is a natural habitat for *Ktedonobacteria*, which may use reduced gases for growth.

These results are consistent with [6], where they found high abundances (37%) of bacteria belonging to the order *Ktedonobacterales* (*Chloroflexi*) in the youngest soil [6]. The carbon fixation pathway in prokaryotes highlighted a predominant function in volcanic soils from the Osorno volcano. *Ktedonobacteria* is reported to uptake CO [57], a potential source of carbon and energy commonly used by microorganisms that colonize volcanic rocks.

These findings are consistent with those reported by [10], in which carbon fixation was one of the most represented functions in all the MAGs obtained from volcanic soils of Chile. However, there are practically no functional differences between NI and H sites.

Analysis of the co-occurrence networks revealed a marked reconfiguration of microbial associations under human influence. The non-intervened soils contained more taxa and links, whereas the humanized soils exhibited a higher positive-to-negative ratio (5.12 vs. 3.15). Because co-occurrence edges can reflect shared environmental filters as much as biotic interactions, these patterns must be interpreted with caution [58,59,60]. Nevertheless, the combination of reduced connectivity and increased positive bias in humanized soils is consistent with stronger environmental filtering and diminished niche partitioning. At the network level, this filtering was reflected in the reduction of negative associations, as shared taxa tended to lose antagonistic links while maintaining positive ones. Genera such as *Sphingomonas* and *Blastocatella* shifted toward exclusively positive profiles, while lineages like *JG30-KF-CM66* (*Chloroflexi*) and *WPS-2* showed higher positive-to-negative ratios due to the decline of negative links. Comparable trends have been documented along land-use intensification gradients, where reductions in negative associations indicate lower niche regulation and reduced dynamical stability [61,62]. In broader anthropogenic contexts, this shift toward positively biased networks reflects the simplification of microbial associations under human disturbance [63,64,65].

The analysis of the functional predictions with PICRUST2 reveals a clear divergence between the undisturbed (NI) and human-impacted (H) volcanic soils in their ecological roles. NI communities are functionally geared towards fundamental biogeochemical processes, possessing pathways for autotrophic carbon fixation, atmospheric trace gas oxidation, and nutrient cycling (C, N, P, S transformations), along with stress tolerance mechanisms. Such traits are characteristic of pioneer microbes in nutrient-poor volcanic environments, where trace gas metabolism (e.g., CO and H_2_ oxidation) can fuel chemosynthesis and initiate soil formation in fresh deposits [66,67]. In contrast, H soils were enriched in genes for carbohydrate catabolism (reflecting reliance on abundant organic inputs) and for competition/stress defenses (e.g., Type III/VI secretion systems, mycothiol biosynthesis). Notably, H communities also harbored more antibiotic efflux pumps, heavy metal resistance determinants, and aromatic compound dioxygenases, indicating adaptation to anthropogenic contaminants [68,69,70]. The prevalence of these pollutant-response functions in H, coupled with the reduced representation of core nutrient-cycling genes, suggests that human disturbance shifted the microbial functional profile from sustaining ecosystem processes toward coping with environmental stressors. This shift is evidenced by the differential abundance analysis: almost all identified soil-health-related KOs (key genes in C, N, P, and O cycles) were enriched in NI, whereas only a few appeared in H (Table S6). Together, these findings imply that anthropogenic pressures may compromise the natural biogeochemical functioning of volcanic soil microbiomes, enhancing stress-tolerance traits at the expense of beneficial ecosystem services.

With respect to modularity, both networks retained this property, with positive associations predominating within modules and negative associations enriched between them, a configuration linked to robustness because it helps to localize perturbations [59,71,72]. However, the humanized soils showed a reduction of negative inter-module associations, suggesting a relaxation of stabilizing competitive checks and stronger covariance across modules. Similar weakening of inter-module antagonism has been observed in volcanic soils with different levels of disturbance and in intensively managed agricultural systems, where the loss of inter-module antagonism coincides with lower buffering capacity [62,73,74].

Finally, the analysis of putative keystone taxa, defined as nodes in the top 10% eigenvector centrality at abundances below the median, revealed greater phylogenetic breadth and central embedding in non-intervened soils, including *Acidobacteriota*, *WPS-2*, and *Gemmatimonadota*, compared with fewer and more peripheral candidates in humanized soils, mainly *Actinobacteriota* and *Chloroflexi*. Although co-occurrence analysis cannot establish causality, rare but highly connected nodes are structurally positioned to stabilize assemblages [11,71]. Shifts in keystone identity under disturbance, favoring stress-tolerant or opportunistic hubs in agricultural, urban, and volcanic systems, have been widely reported [61,63,73,74]. Taken together, the contraction of interactional diversity, the reduction of inter-module antagonism, and the narrowing of keystone breadth in humanized soils indicate a transition toward facilitation-dominated, environmentally filtered networks that may appear cohesive but with lower buffering capacity and resilience.

## 5. Conclusions

In this study, we identified the structure of bacterial communities in volcanic soils in northern Chilean Patagonia. Overall, the results of the study revealed significant differences in alpha and beta diversity indices between areas with and without anthropogenic activities. Specifically, four phyla and 105 families exhibited substantial shifts in their relative abundance between the two regions, indicating that members of these taxa may exhibit particular associations with the abiotic (physicochemical soil composition) and biotic (vegetation composition) conditions associated with increased anthropogenic activity. However, it must be recognized that changes in abiotic or biotic factors could also be natural in origin, thus obscuring the impact of anthropogenic activity. Additional field studies are necessary to differentiate between these possibilities.

## Figures and Tables

**Table 1:** Summary of 16S rRNA gene sequencing, ASVs, richness, and diversity.

Sample Internal Code	Type of Area	Assigned Sequences	Total ASVs (Richness)	Chao1 Index	Shannon Index	Phylogenetic Diversity (PD)	Good’s Coverage	NCBI Accession
H1.1	Humanized	68,525	947	1,032	8	198	99.5	SAMN35159685
H1.2	Humanized	63,869	1,193	1,255	8.8	217	99.6	SAMN35159686
H1.3	Humanized	63,284	1,841	2,015	8.8	304	99	SAMN35159687
H2.1	Humanized	55,326	1,836	1,937	9.3	272	99.3	SAMN35159688
H2.2	Humanized	59,035	1,711	1,831	9.1	234	99.2	SAMN35159689
H2.3	Humanized	59,328	1,244	1,334	8	246	99.4	SAMN35159690
H3.1	Humanized	58,607	1,887	2,027	8.8	337	99.1	SAMN35159691
H3.2	Humanized	58,755	1,470	1,537	8.5	282	99.5	SAMN35159692
H3.3	Humanized	60,309	1,561	1,674	8.4	241	99.3	SAMN35159693
NI1.1	Non-intervened	34,563	2,256	2,257	10	356	99.9	SAMN35159694
NI1.2	Non-intervened	33,802	2,383	2,383	10.1	312	99.9	SAMN35159695
NI2.1	Non-intervened	53,979	3,188	3,350	10.5	371	98.7	SAMN35159696
NI2.2	Non-intervened	54,642	3,232	3,418	10.6	352	98.6	SAMN35159697
NI2.3	Non-intervened	58,711	3,862	4,185	10.9	510	97.9	SAMN35159698
NI3.1	Non-intervened	53,866	3,308	3,511	10.3	459	98.4	SAMN35159699
NI3.2	Non-intervened	57,031	3,607	3,856	10.6	429	98.2	SAMN35159700
NI3.3	Non-intervened	59,409	3,436	3,626	10.6	447	98.6	SAMN35159701

## Data Availability

16S rRNA gene sequencing data have been submitted to the NCBI BioProject database under accession number PRJNA974056.

## Supplementary Materials

The following supporting information can be downloaded at: https://www.mdpi.com/article/10.3390/microorganisms13112519/s1, Figure S1: Map of sampling sites; Figure S2: Bray-Curtis similarity analysis (dendrogram) using the relative abundance of all ASVs in all volcanic soil samples. Green and red colors correspond to locations: non-intervened (NI) and humanized (H), respectively; Figure S3: Heatmap and samples clustering based on the relative abundance of metabolism pathways based on KEGG orthologous gene identification by PICRUSt2 analysis; Table S1: Sampling points, GPS coordinates and types of area. The size of the sampling area was 1 square meter per sample; Table S2: Physicochemical characteristics of the volcanic soils (compound samples) of the Osorno Volcano, Los Lagos region, Chile; Table S3: Concentrations of trace elements (mg/Kg) in the analyzed volcanic soil samples. Table S4: Ribosomal 16S amplicon sequence variants (ASV) frequency after the rarefaction to 60.000 reads; Table S5: Family level relative abundance (percentage) for each sample. * indicate Class; Table S6: Abundance (percentage) of KOG predicted functions for each sample at different KOG description levels; Table S7: Summary of network topology and interaction patterns in NI and H co-occurrence networks.
