# Supplementary material for: Characterization of Bacterial Communities in Volcanic Soil from Northern Patagonian Area of Chile
Source: Microorganisms. 2025 Nov 1;13(11):2519. doi: 10.3390/microorganisms13112519 (PMC12654796; doi:10.3390/microorganisms13112519)
Supplement: Supplementary file 1 [file microorganisms-13-02519-s001.zip › Table S1.pdf]

Table S1. Sampling points, GPS coordinates and types of area. The size of the sampling area was 1 square meter per sample.

Site	Type of area	Coordinates (latitude)	Coordinates (longitude)
H1.1	humanized	41°12'49.02"S	72°53'16.25"W
H1.2	humanized	41°12'49.02"S	72°53'16.25"W
H1.3	humanized	41°12'49.02"S	72°53'16.25"W
H2.1	humanized	41°12'70.36"S	72°53'08.57"W
H2.2	humanized	41°12'70.36"S	72°53'08.57"W
H2.3	humanized	41°12'70.36"S	72°53'08.57"W
H3.1	humanized	41°12'69.12"S	72°53'07.97"W
H3.2	humanized	41°12'69.12"S	72°53'07.97"W
H3.3	humanized	41°12'69.12"S	72°53'07.97"W
NI1.1	Non-intervened	41°13'20.15"S	72°53'33.04"W
NI1.2	Non-intervened	41°13'20.15"S	72°53'33.04"W
NI2.1	Non-intervened	41°13'22.69"S	72°53'38.76"W
NI2.2	Non-intervened	41°13'22.69"S	72°53'38.76"W
NI2.3	Non-intervened	41°13'22.69"S	72°53'38.76"W
NI3.1	Non-intervened	41°13'41.12"S	72°53'46.38"W
NI3.2	Non-intervened	41°13'41.12"S	72°53'46.38"W
NI3.3	Non-intervened	41.13'41.12"S	72°53'46.38"W
