# Supplementary material for: Characterization of Bacterial Communities in Volcanic Soil from Northern Patagonian Area of Chile
Source: Microorganisms. 2025 Nov 1;13(11):2519. doi: 10.3390/microorganisms13112519 (PMC12654796; doi:10.3390/microorganisms13112519)
Supplement: Supplementary file 1 [file microorganisms-13-02519-s001.zip › Table S2.pdf]

Table S2. Physicochemical characteristics of the volcanic soils (compound samples) of the Osorno Volcano, Los Lagos region, Chile.

**Sites**	**Humanized**		**Non-intervened**	
Particle size (%)	Clay	6	Clay	8
	Silt	16	Silt	22
	Sand	78	Sand	70
pH in suspension	7.0		6.9	
Electric conductivity (mS/cm)	0.06		0.10	
Organic matter (%)	0.10		0.89	
N (%)	0.02		0.05	
C (%)	0.06		0.52	
Sieve >4 mm %	30.26		27.48	
Sieve >2 mm %	10.90		15.68	
Sieve >1 mm %	16.05		20.63	
Sieve >0.075 %	36.81		29.48	
Remaining sieve %	5.98		6.74	
