# Supplementary material for: Characterization of Bacterial Communities in Volcanic Soil from Northern Patagonian Area of Chile
Source: Microorganisms. 2025 Nov 1;13(11):2519. doi: 10.3390/microorganisms13112519 (PMC12654796; doi:10.3390/microorganisms13112519)
Supplement: Supplementary file 1 [file microorganisms-13-02519-s001.zip › Table S3.pdf]

Table S3. Concentrations of trace elements (mg/Kg) in the analyzed volcanic soil samples.

Parameters (mg kg^{-1})	Abbreviation	H1.1+H1.2+H1.3 average	H2.1+H2.2+H2.3 average	H3.1+H3.2+H3.3 average	NI1.1+NI1.2+NI1.3 average	NI2.1+NI2.2+NI2.3 average	NI3.1+NI3.2+NI3.3 average
Arsenic	As	0,494	0,091	0,038	0,089	0,086	0,125
Cadmium	Cd	0,55	0,823	0,675	0,549	0,699	0,424
Copper	Cu	7,2	61	68	9,2	4	6,67
Chrome	Cr	1,73	1,97	1,5	1,56	0,674	1,07
Iron	Fe	2374	4371	2368	2570	1652	2242
Manganese	Mn	36	70	50	46	21	30
Mercury	Hg	0,077	0,076	0,152	0,061	0,056	0,062
Molybdenum	Mo	< 4,5	< 4,5	< 4,5	< 4,5	< 4,5	< 4,5
Nickel	Ni	5,33	6,36	6,1	5,39	4,5	4,57
Lead	Pb	< 0,5	6,96	5,67	< 0,5	< 0,5	< 0,5
Selenium	Se	0,012	0,021	0,014	0,012	0,016	0,017
Zinc	Zn	3,62	6,56	4,2	8,01	2,35	4,99
