# Supplementary material for: Characterization of Bacterial Communities in Volcanic Soil from Northern Patagonian Area of Chile
Source: Microorganisms. 2025 Nov 1;13(11):2519. doi: 10.3390/microorganisms13112519 (PMC12654796; doi:10.3390/microorganisms13112519)
Supplement: Supplementary file 1 [file microorganisms-13-02519-s001.zip › Title supl figures and tables.pdf]

Table S1. Sampling points, GPS coordinates and types of area. The size of the sampling area was 1 square meter per sample.

Table S2. Physicochemical characteristics of the volcanic soils (compound samples) of the Osorno Volcano, Los Lagos region, Chile.

Table S3. Concentrations of trace elements (mg/Kg) in the analyzed volcanic soil samples.

Table S4. Ribosomal 16S amplicon sequence variants (ASV) frequency after the rarefaction to 60.000 reads.

Table S5. Family level relative abundance (percentage) for each sample. * indicate Class.

Table S6. Abundance (percentage) of KOG predicted functions for each sample at different KOG description levels.

Table S7. Summary of network topology and interaction patterns in NI and H co-occurrence networks.

Figure S1. Map of sampling sites.

Figure S2. Bray-Curtis similarity analysis (dendrogram) using the relative abundance of all ASVs in all volcanic soil samples. Green and red colors correspond to locations: non-intervened (NI) and humanized (H), respectively.

Figure S3. Heatmap and samples clustering based on the relative abundance of metabolism pathways based on KEGG orthologous gene identification by PICRUSt2 analysis. Only samples with an average of at least 0.5 percent abundance are represented. Samples (columns) were clustered using the one minus pearson correlation and pathways (rows) were clustered using the Euclidean distance, and in both cases there were used the average linkage method. Abundance is represented as a ratio between 0 and 1. This figure was prepared with the online tool “Morpheus” of the Broad Institute (https://software.broadinstitute.org/morpheus/).
