# Supplementary figures and images for: Characterization of Bacterial Communities in Volcanic Soil from Northern Patagonian Area of Chile
Source: Microorganisms. 2025 Nov 1;13(11):2519. doi: 10.3390/microorganisms13112519 (PMC12654796; doi:10.3390/microorganisms13112519)

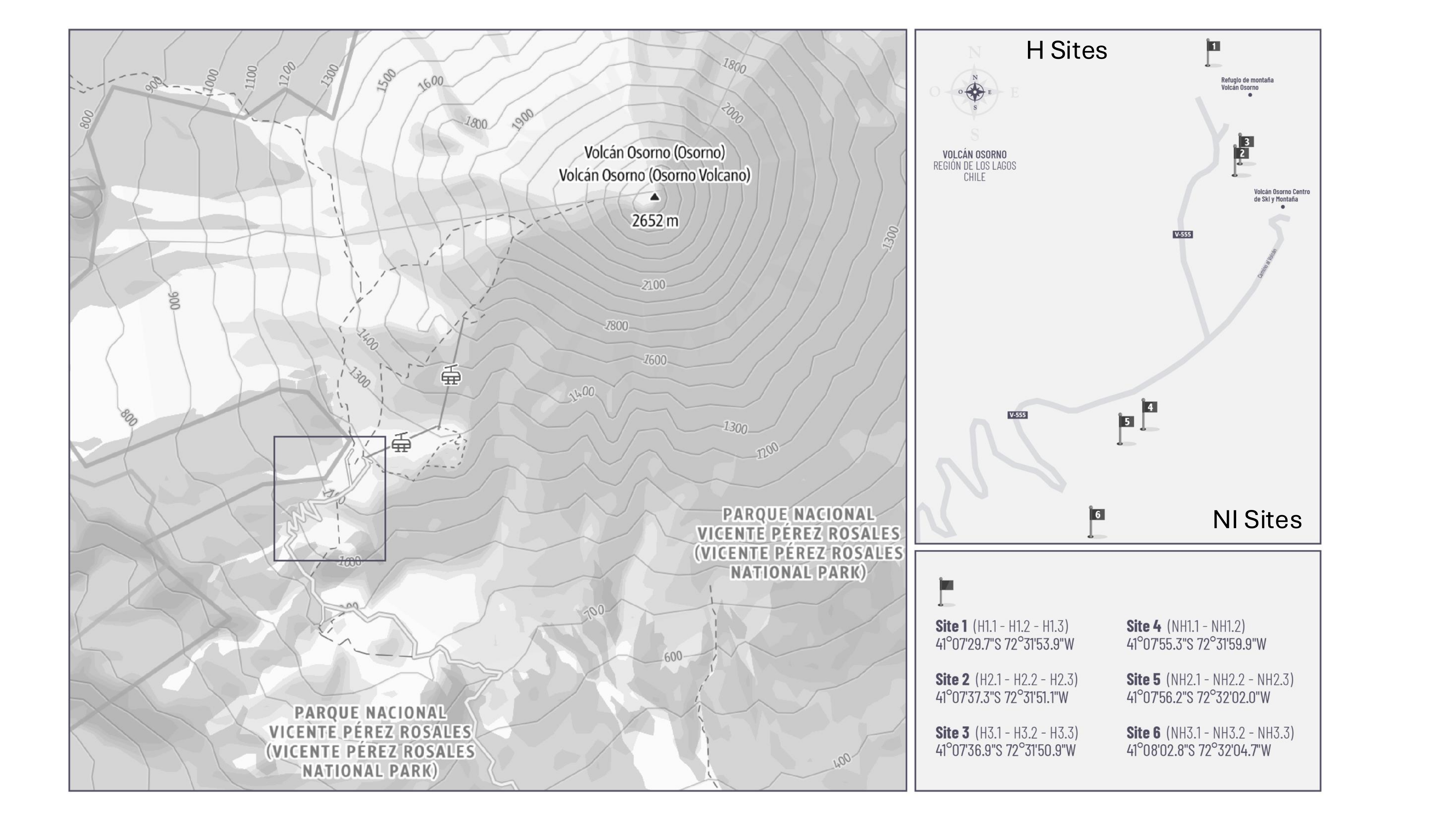

Supplement: Supplementary file 1 [file microorganisms-13-02519-s001.zip › Figure S1.tif]

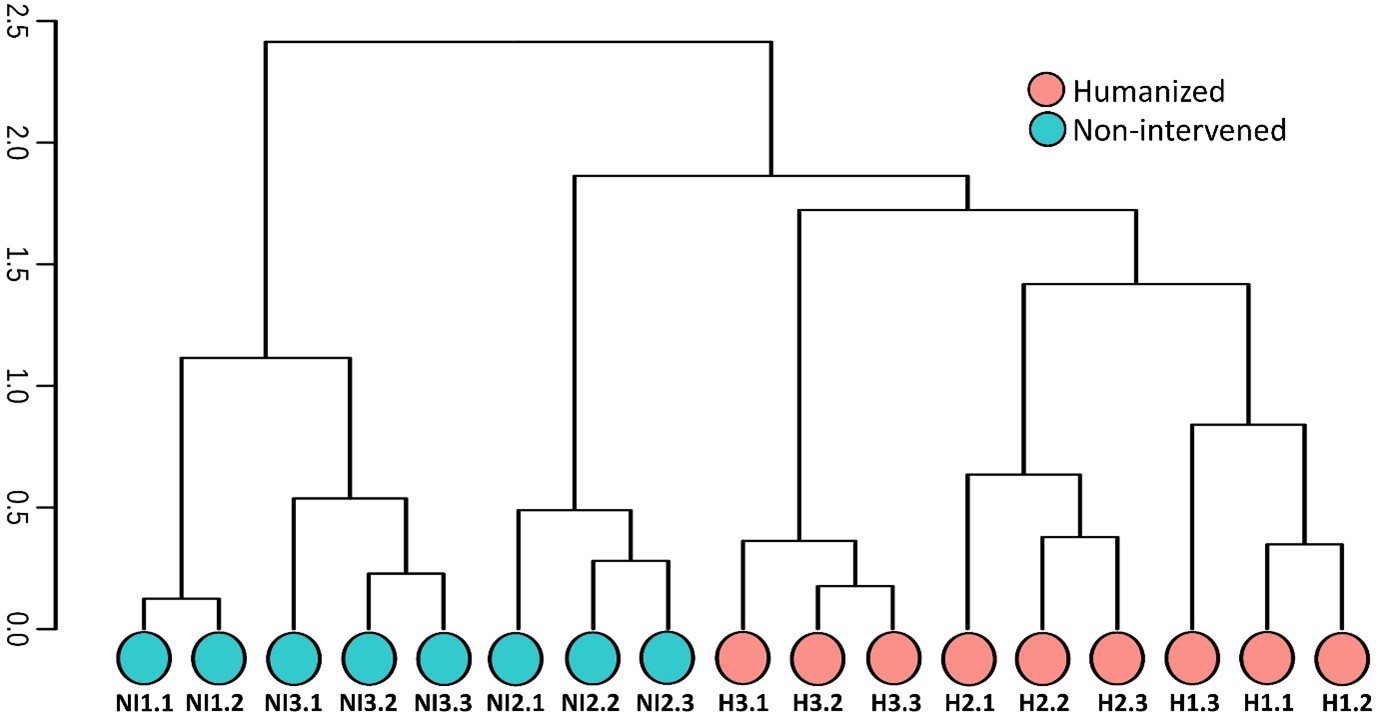

Supplement: Supplementary file 1 [file microorganisms-13-02519-s001.zip › Figure S2.jpg]

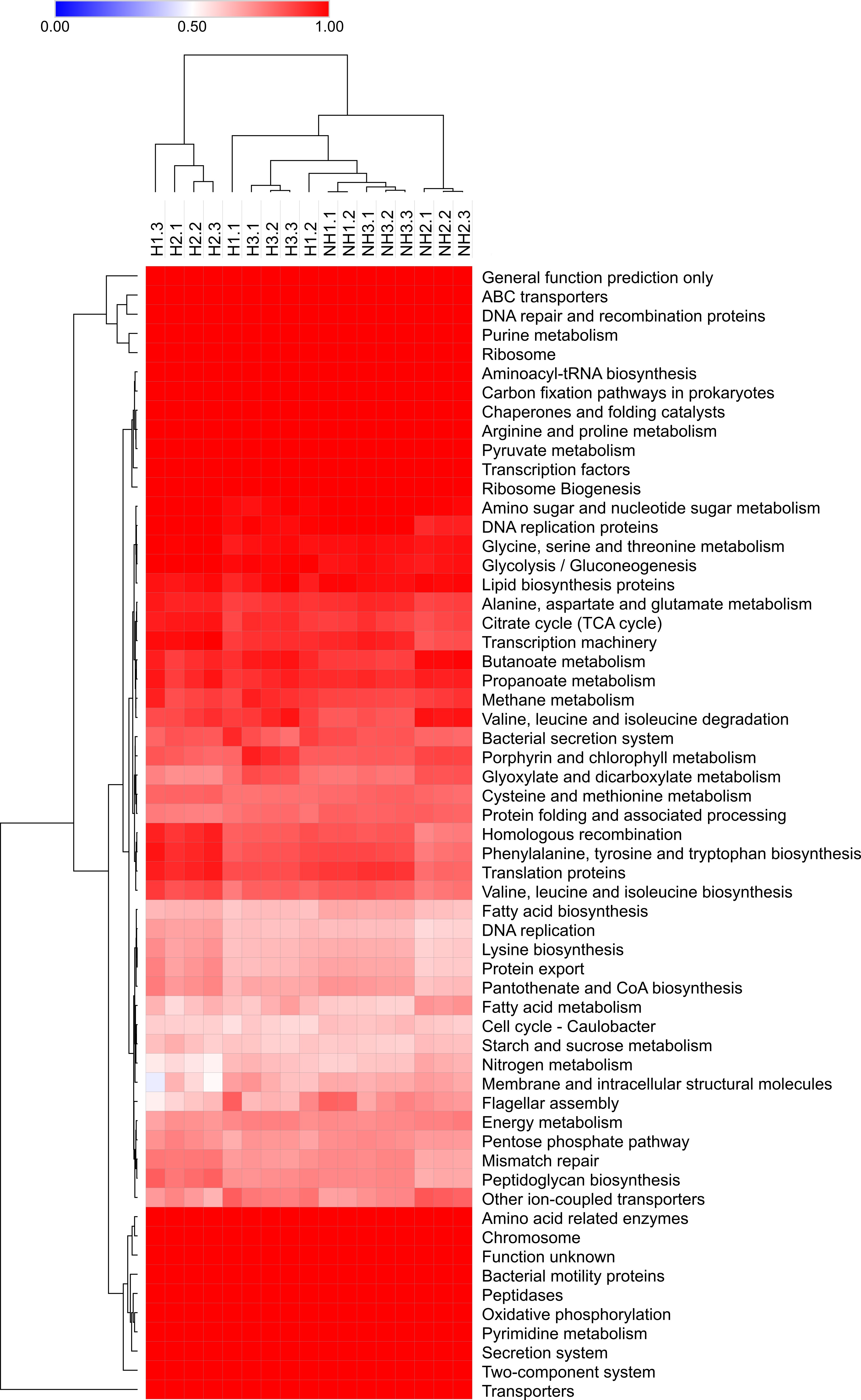

Supplement: Supplementary file 1 [file microorganisms-13-02519-s001.zip › Figure S3.jpg]
